# A Spatial Analysis of Atmospheric Ammonia and Ammonium in the U.K.

**DOI:** 10.1100/tsw.2001.313

**Published:** 2001-11-28

**Authors:** M.A. Sutton, Y.S. Tang, U. Dragosits, N. Fournier, A.J. Dore, R.I. Smith, K. J. Weston, D. Fowler

**Affiliations:** Center for Ecology and Hydrology, Edinburgh Research Station, Bush Estate, Penicuik, UK

**Keywords:** acidification, eutrophication, aerosol, monitoring, atmospheric transport model, deposition

## Abstract

As measures are implemented internationally to reduce SO_2_ and NO_x_ emissions, attention is falling on the contribution of NH_3_ emissions to acidification, nitrogen eutrophication, and aerosol formation. In the U.K., a monitoring network has been established to measure the spatial distribution and long-term trends in atmospheric gaseous NH_3_ and aerosol NH_4_. At the same time, an atmospheric chemistry and transport model, FRAME, has been developed with a focus on reduced nitrogen (NH_x_). The monitoring data are important to evaluate the model, while the model is essential for a more detailed spatial assessment. The national network is established with over 80 sampling locations. Measurements of NH_3_ and NH_4_ (at up to 50 sites) have been made using a new low-cost denuder-filterpack system. Additionally, improved passive sampling methods for NH_3_ have been applied to explore local variability. The measurements confirm the high spatial variability of NH_3_ (annual means 0.06 to 11 mg NH_3_ m), consistent with its nature as a primary pollutant emitted from ground-level sources, while NH_4_, being a slowly formed secondary product, shows much less spatial variability (0.14 to 2.4 mg NH_4_ m). These features are reproduced in the FRAME model, which provides estimates at a 5-km level. Analysis of the underlying NH_3_ emission inventory shows that sheep emissions may have been underestimated and nonagricultural sources overestimated relative to emissions from cattle. The combination of model and measurements is applied to estimate spatial patterns of dry deposition to different vegetation types. The combined approach provides the basis to assess NHx responses across the U.K. to international emission controls.

## INTRODUCTION

Atmospheric ammonia ($NH_3$) is of interest because of its effects on atmospheric chemistry and on seminatural ecosystems. In the atmosphere, $NH_3$ reacts to form ammonium ($NH_4^+$) aerosol, which has a negative effect on radiative forcing, reduces visibility, and may have negative effects on human health. In addition, the formation of $NH_4^+$ affects the atmospheric transport distance of $SO_2$ and $NO_x$ emissions, while $NH_4^+$ salts are the vector for most of the transboundary transport and deposition of atmospheric acidity[1,2]. Once deposited, $NH_4^+$ releases acidity, since the nitrogen is either accumulated in organic R-$NH_2$ forms (1 $H^+$ produced, originating from $SO_2$ or $NO_x$) or nitrified and leached as $NO_3^-$ (producing 2 $H^+$, including one $H^+$ from $NH_3$ itself)[2]. In addition to the acidifying effect, the input of nitrogen from either

$NH_3$ or $NH_4^+$ (collectively $NH_x$) leads to eutrophication of seminatural habitats, which may result in species composition changes[3].

As with other atmospheric pollutants, $NH_3$ emissions have increased substantially in the 20th century[4,5], and there is currently international momentum to set policy measures to reduce these emissions. For example, under the UNECE Convention on Long-Range Transboundary Air Pollution, international ceilings of $NH_3$ emissions have been set for the first time with the 1999 Gothenburg Protocol. Within the context of expected ecological effects and investigation of the costs of emission abatement, it is essential to have a sound quantification of the concentrations and fluxes of $NH_x$ in the atmosphere. Since neither the distribution of $NH_3$ emissions nor the distribution of sensitive habitats is uniform, it is necessary to assess the spatial distribution of $NH_x$ concentrations and deposition. Complementary to this is the need to be able to monitor long-term trends in order to quantify the link to emission changes over periods of several years.

Monitoring the complete distribution of $NH_3$ concentrations at a national level would require an impracticably large (>1000) number of stations, due to the spatial variability characteristic of a primary pollutant emitted at or near ground level[6,7]. Atmospheric transport models therefore serve a complementary role, since, using spatially resolved emission estimates, national maps of predicted $NH_3$ concentrations may be established at, e.g., 5-km resolution[8,9]. By treating the chemical reactions with other pollutants in such models, they may also be used to assess the interactions with and distribution of $NH_4^+$ aerosol and wet deposition. A finding of models of this kind is that the distribution of $NH_4^+$ aerosol is expected to be less spatially variable, since it is formed over the course of minutes to hours as a secondary atmospheric product[10].

Most monitoring data for $NH_x$ in the atmosphere are available for $NH_4^+$ in precipitation, but there is much less monitoring information available for speciated measurements of gaseous $NH_3$ and $NH_4^+$ aerosol[5]. One of the reasons for this is the need for active sampling to distinguish the gas and aerosol, which is either cumbersome or expensive. Similarly, although methods have been reported for passive sampling of $NH_3$ (see review[11]), these have experienced mixed success, with, in some cases, substantial and uncertain correction factors needing to be applied to data.

To address these problems in the U.K., a new low-cost denuder-filterpack system was developed tuned for long-term sampling. The method, referred to as the DELTA (DEnuder for Long-Term Ammonia)[12], allows sampling with 2-weekly or monthly duration and operates robustly for concentrations in the range 0.01 to >40 $\mu g\ m^{-3}$. In this approach, $NH_3$ is collected in two acid-coated glass denuders in series, while $NH_4^+$ aerosol is captured on a subsequent acid-coated paper filter. In parallel, improvements in $NH_3$ passive sampling have been made to allow reliable implementation. First, the performance of 35-mm path-length diffusion tubes has been improved to permit monthly measurements down to a level of c.1 $\mu g\ m^{-3}$[11,13]. Second, the ALPHA (Adapted Low-cost Passive High Absorption) sampler with 6-mm path length was developed[14], which is able to sample down to <0.05 $\mu g\ m^{-3}$ with a monthly sampling period.

In this article, we report the application of these new sampling methods in the U.K. National Ammonia Monitoring Network (NAMN)[15] and show how these compare with estimates from the Fine Resolution AMmonia Exchange (FRAME) atmospheric dispersion model[9]. Combined with a disaggregation of $NH_3$ emissions data, the results are used to compare areas of the U.K. dominated by different major source sector types. Finally, the combination of the measurements and modeling is used to estimate the spatial patterns of $NH_3$ dry deposition across the U.K. and to consider the implications of the results for environmental policy.

## METHODS

### Monitoring Network Strategy

The NAMN was established to provide the best-measured $NH_3$ concentration field for the U.K. and at the same time to provide data for the assessment of models. Given the expense of high time-resolution monitoring[16,17], the strategy for the network was to sample at a large number of sites to assess spatial variability, using low-frequency sampling to allow long-term trends to be assessed at low cost. This strategy of high network density and low sampling frequency contrasts with the alternatives of hourly or daily monitoring[16], which would permit sampling at only a very few (2 to 4) sites with similar resources. A total of 120 potential sites were identified for consideration, and the following criteria for selection were identified[15]:

a. Representative spatial coverage of the U.K.
b. Representative coverage in relation to predicted FRAME $NH_3$ concentrations at a 5-km grid level, including key emission peak and trough areas
c. Relationship of the sites to local $NH_3$ sources (avoiding being <150 m from point sources)
d. Availability and access to a willing and helpful local contact
e. Availability of mains electricity at a site (for DELTA sampling)
f. Co-location of the sites in relation to other air-quality and ecosystem-monitoring activities
g. Nature conservation interest of the site, particularly in relation to plant community sensitivity to nitrogen deposition and nature reserve status.

On the basis of these criteria, and following establishment of the sampling methods[11], the network was established with 70 sites in 1996, with DELTA sampling at 50 sites, diffusion tube sampling at 30 sites, and both methods implemented at 10 sites. Following a review of the network in 1998, changes were made, resulting in a total of 80 sites and introduction of $NH_4^+$ sampling. DELTA sites were distributed widely across the U.K. to provide regional patterns of $NH_3$ and $NH_4^+$, while passive sampling concentrated on the assessment of mesoscale variability in source areas as a test of the $NH_3$ emission-dispersion modeling (Fig. 1). Three mesoscale study areas were identified: Welsh Borders/Shropshire, the Norfolk/Suffolk border, and the Yorkshire Vale. An absence of mains electricity at three active sampling sites led to the development of three solar-wind-powered DELTA systems[12]. Data are reported here up to November 2000, starting in September 1996 for $NH_3$ and April 1999 for $NH_4^+$.

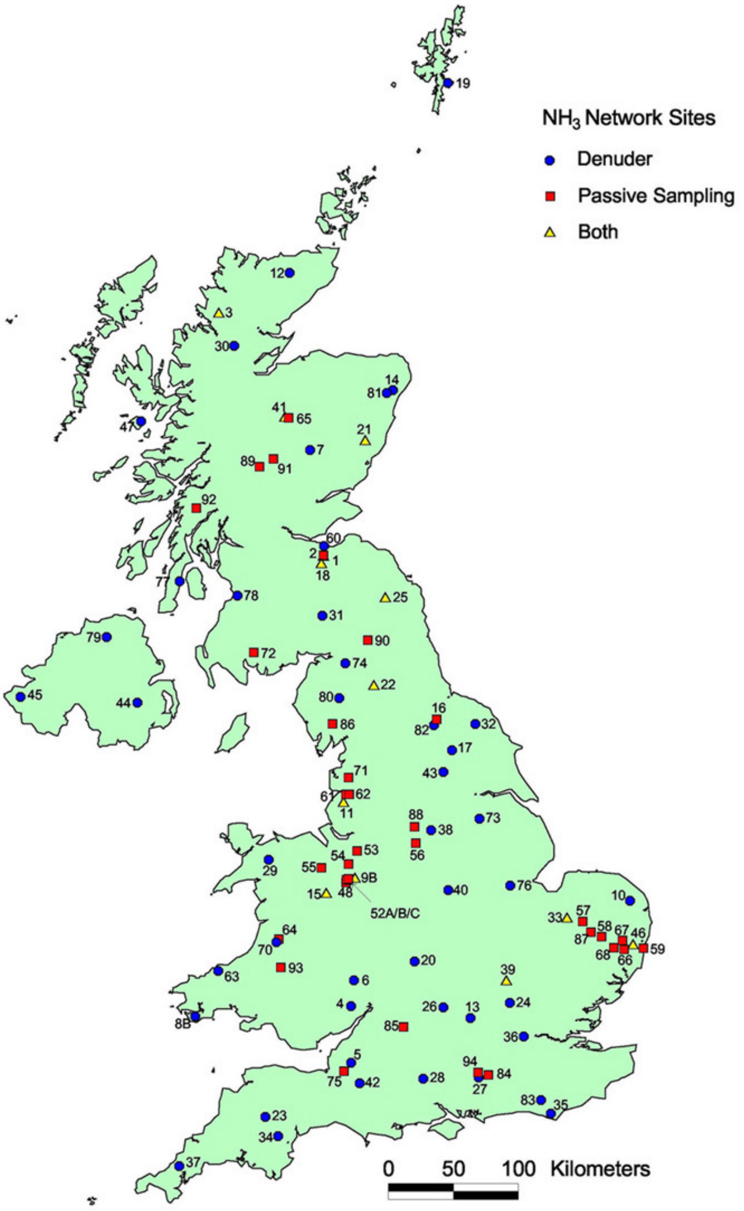

**FIGURE 1.** Map of sites in the U.K. National Ammonia Monitoring Network using either denuders, triplicate diffusion tubes, or both. (Site numbers are explained in the Appendix.)

Chemical analysis of aqueous extracts from each of the DELTA and passive sampling methods was performed using flow injection analysis with selective membrane diffusion of $NH_3$ at high pH and detection of $NH_4^+$ by conductivity[18].

## Emission and Atmospheric Transport Modeling

The distribution of $NH_3$ emissions for the U.K. is modeled in a GIS methodology described by Dragosits et al.[19] For agricultural emissions, parish statistics on livestock numbers and crop areas are combined with satellite-based land-cover data to model emissions at a 1-km resolution, which are subsequently aggregated to the 5-km level. Spatial inventories have been established with this approach for 1988 and 1996.

The FRAME atmospheric chemistry and dispersion model[9,20] is run using the disaggregated $NH_3$ emissions together with $SO_2$ and $NO_x$ emissions. FRAME is a statistical Lagrangian dispersion model, which employs straight trajectories and a multilayer dispersion scheme[21] to simulate the vertical profiles of trace gases. In addition, a land-cover-dependent dry deposition scheme is applied for $NH_3$. In the model version applied here, dry deposition is limited by characteristic canopy resistances ($R_c$) set for different vegetation types[9]. It should be noted that the domain of FRAME covers both Britain and Ireland, although results are presented here only for the U.K., which includes Great Britain and Northern Ireland.

The spatial distribution of $NH_3$ emission source sectors is not uniform across the country. Hence, one of the advantages of a network with many sites is that areas with emissions dominated by different source sectors can be compared. To do this, the spatial $NH_3$ inventory was analyzed to show areas where >45% of the emission was due to a given source. Areas of background emission (<1 kg N $ha^{-1}$ $year^{-1}$) were distinguished, while pig and poultry farms were combined to allow improved visualization and comparison between nonland-based and land-based farming. The resulting map of dominant $NH_3$ source sectors is shown in Fig. 2. Due to disclosivity issues regarding the data for 1996, Fig. 2 shows the results for 1988, although the patterns for the 2 years are similar.

## Deposition Modeling

While the FRAME model provides estimates of the dry deposition field for different vegetation types and with a resolution of 5 km, deposition estimates derived directly from the measurements would be considered more reliable. In the first instance, dry deposition fields may be estimated using the interpolated measured $NH_3$ concentrations and an inferential resistance model. However, this implies large assumptions in the interpolation of con-

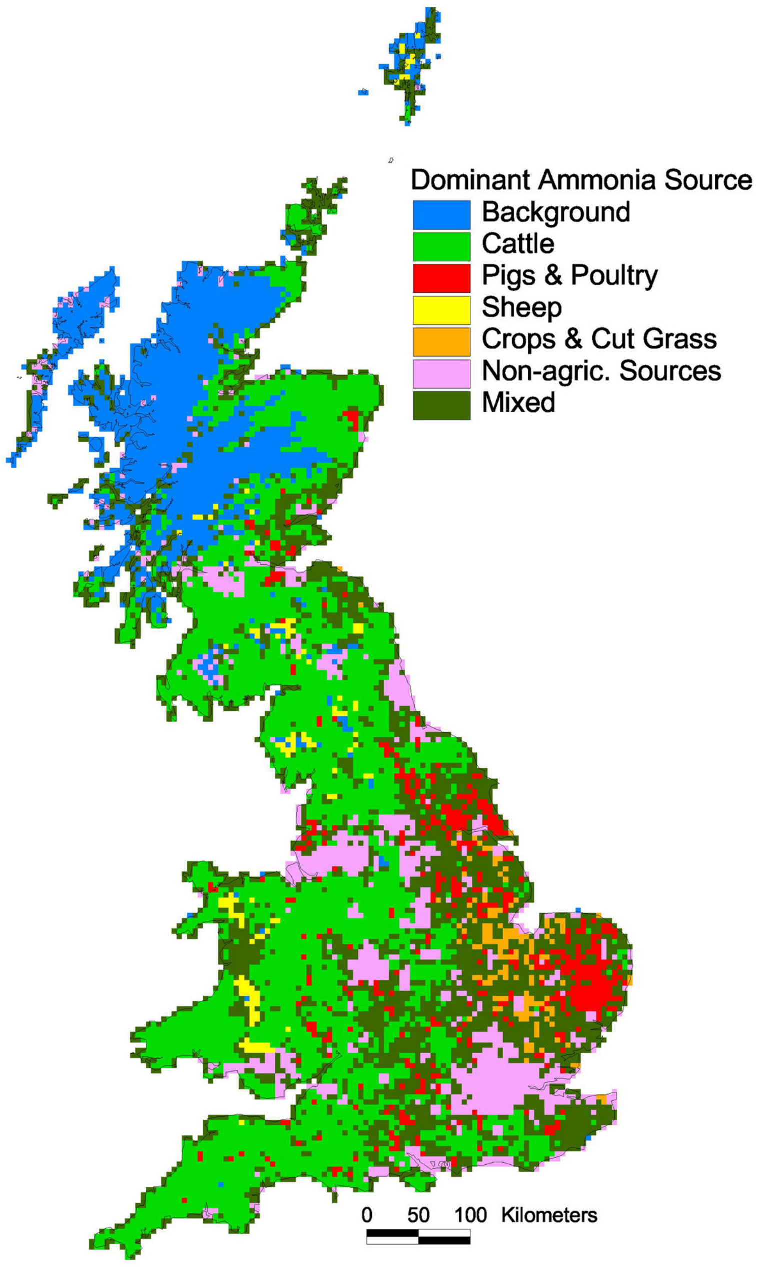

**FIGURE 2.** Classification of 5-km grid resolution $NH_3$ emissions for Great Britain (Northern Ireland not included) according to estimated dominant (>45% contribution) source sector. Background is set here at <1 kg N $ha^{-1}$ $year^{-1}$.

centrations. Unless the dispersion model provides a perfect fit to the measurements, an alternative approach is therefore to calibrate the dispersion model output concentration estimates, and this method has been applied here. The regression between the mean NAMN concentrations and the FRAME-predicted $NH_3$ concentrations at the same sites (see Figure 5a) is applied to the FRAME estimates for each grid square in the U.K. to provide a revised $NH_3$ concentration field. It should be noted that this is merely a calibration of model output, rather than an internal change to the model. The revised $NH_3$ concentration field is then applied as input in the inferential model of Smith et al.[22]

## RESULTS AND DISCUSSION

### Measured $NH_3$ and $NH_4^+$ Concentrations

Overall, the mean concentrations in the network for the sampling periods reported above were in the range 0.06 to 11 μg $NH_3$ $m^{-3}$ and 0.14 to 2.4 μg $NH_4^+$ $m^{-3}$ (Fig. 3). A summary of the data is given in the Appendix. In the case of $NH_3$, much larger concentrations were seen in source areas, particularly in areas with intensive cattle, pig, and poultry farming, while, within only a few tens of kilometers, much smaller concentrations were found in areas with few $NH_3$ sources. For example, at North Wyke in Devon (site 23), the mean concentration was 1.71 μg $m^{-3}$, while 25 km southeast the concentration at Yarner Wood (site 34) was 0.52 μg $m^{-3}$. This high level of spatial variability is seen even more in the mesoscale variability sampling results. The measurements in Norfolk/Suffolk cover both intensive farming and sink areas and show mean concentrations in the range 1.23 (Dunwich Heath, site 59) to 10.7 μg $m^{-3}$ (Bedlingfield, site 68) with gradients of up to 0.4 μg $NH_3$ $m^{-3}$ $km^{-1}$ over distances of 10 to 20 km. This illustrates the substantial local spatial variability of $NH_3$ concentrations, even when sampling in the vicinity of point sources is avoided. Even larger gradients occur adjacent to point sources[7,23], making it difficult to assess regional-level variability of $NH_3$ concentrations, and it was for this reason that sampling near known point sources was avoided.

By contrast, $NH_4^+$ aerosol showed much less local spatial variability, with a smooth interpolated field decreasing from 2.4 μg $m^{-3}$ in central London (site 36c) and 2.2 μg $m^{-3}$ in a high $SO_2$ emission area of northern England (Jenny Hurn, site 73) to 0.14 μg $m^{-3}$ at Rum on the northwest coast of Scotland (site 47). This reflects the nature of $NH_4^+$ aerosol as a secondary product that is produced throughout the boundary layer. By sampling with a large number of sites, it was hypothesized that it should be possible to demonstrate this expected smooth pattern, since adjacent sites should have similar concentrations. This is shown in Fig. 3, with the local effects that occur for $NH_3$ generally not being seen. For

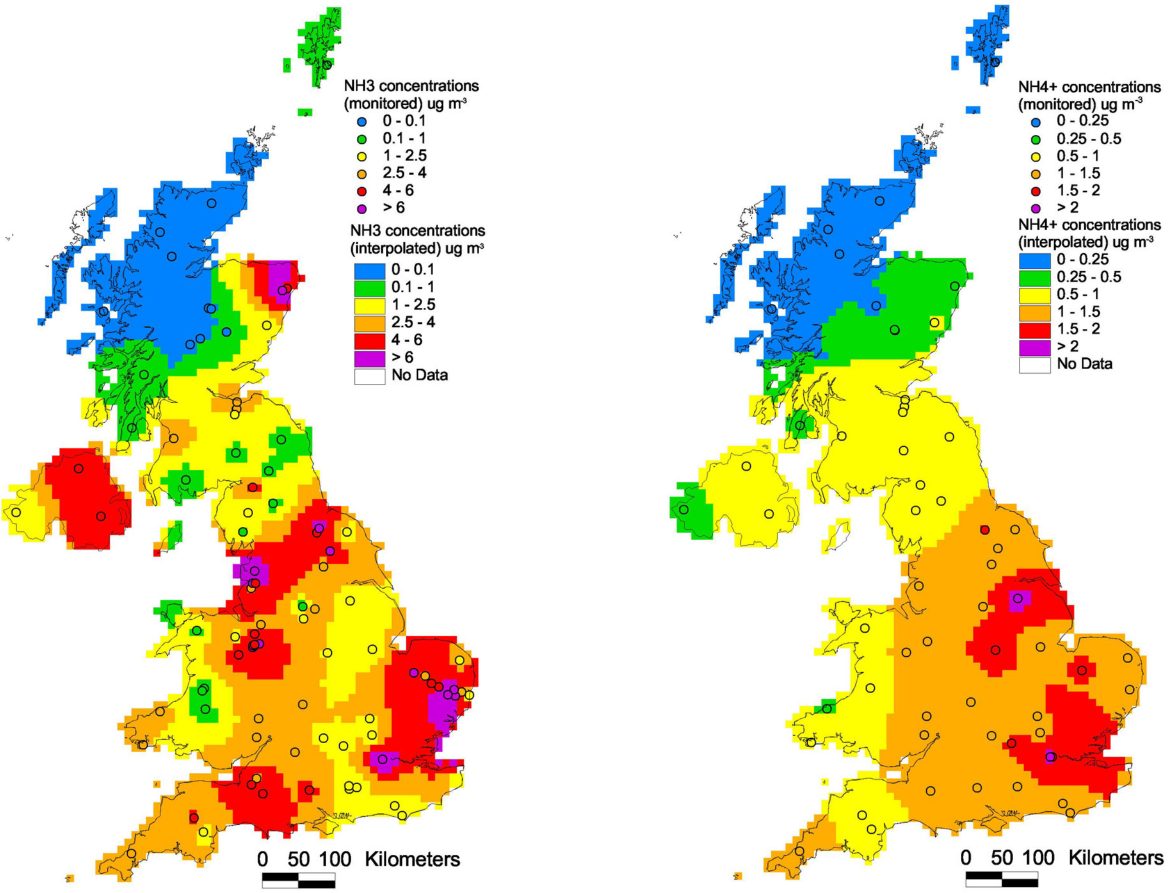

**FIGURE 3.** Interpolated mean measured gaseous $NH_3$ and aerosol $NH_4^+$ concentrations from the U.K. National Ammonia Monitoring Network.

example, the $NH_4^+$ concentrations at North Wyke and Yarner Wood are 0.93 and 0.90 µg $m^{-3}$, respectively. Such small differences between adjacent sites are typical. As a consequence of this, following November 2000, the number of aerosol sampling sites has been reduced to 30, which is sufficient to resolve the main spatial features of the U.K. $NH_4^+$ aerosol field.

## Modeled $NH_3$ Concentrations and Comparison with Measurements

The performance of the FRAME model in predicting average $NH_3$ and $NH_4^+$ concentrations is shown in Fig. 4. As FRAME is a multilayer model, concentrations can be reported for different heights, and the values in Fig. 4 are the average of the layer at 1 to 2 m. Consistent with $NH_3$ emissions arising from ground level, the modeled $NH_3$ concentration field shows substantial spatial variability, and this matches closely to the distribution of $NH_3$ emissions. The other major factors affecting $NH_3$ concentration are the removal rates by wet and dry deposition. Hence for a given emission rate, modeled concentrations of $NH_3$ are higher in eastern rather than western Britain, due to less precipitation scavenging and increased occurrence of agricultural croplands, which experience less dry deposition. The highest modeled concentrations of $NH_3$ occur in areas with intensive pig and poultry farming (East Anglia, Yorkshire), cattle farming (Shropshire, Lancashire, Northern Ireland), and nonagricultural emissions (London) (cf. Fig. 2).

As with the measurements, the spatial pattern of modeled $NH_4^+$ aerosol is much less spatially variable than $NH_3$. The highest concentrations again occur in London and in high $SO_2$ emission areas of northern England, while the lowest concentrations occur in northern and western parts of the U.K.

A comparison of the FRAME predictions with the network measurements for gaesous $NH_3$ and $NH_4^+$ aerosol is shown in Fig. 5. The network average for each site is compared against the model estimate for the 5-km grid square in which it occurs, and the point is classified according to the estimated dominant source sector of the square. Since the concentrations in the network and FRAME are log-normally distributed, Fig. 5 shows the comparison on a logarithmic scale. The first point to note is that the correlation for $NH_4^+$ aerosol ($R^2_{[log\ values]}$ = 0.91; $R^2_{[absolute\ values]}$ = 0.82) is much higher than that for $NH_3$ ($R^2_{[log\ values]}$ = 0.70; $R^2_{[absolute\ values]}$ = 0.57). This may be explained by the large spatial variability of $NH_3$, with the result that it is difficult to monitor at the most representative location in a 5-km grid square. Many of the sites where the model overestimates the measurements are, in fact, nature reserve or forest sites. The reason for the overestimation may therefore be due to subgrid variability not addressed by the model, as the monitoring sites in these sink areas would be more than the average distance from sources in the same 5-km grid square. Conversely, some of the outliers, where the

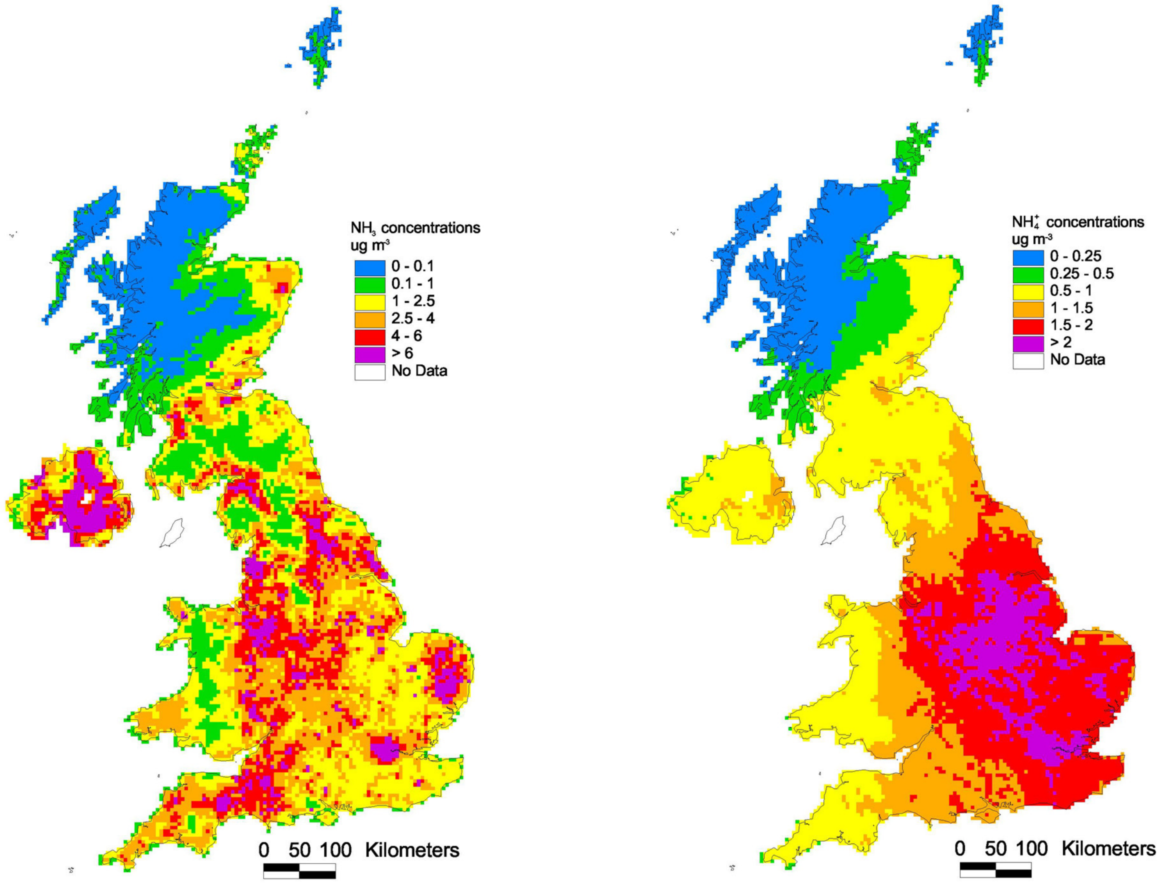

**FIGURE 4.** Distribution of gaseous $NH_3$ and aerosol $NH_4^+$ across the U.K. according to the FRAME model.

measurements are larger than the model, show some indication of being affected by nearby sources. For example, at Glenshee (site 7, emissions dominated by cattle) in the Scottish highlands, the measurement estimate was 2.66 µg m$^{-3}$, while FRAME estimated 0.28 µg m$^{-3}$. The 5-km grid square in question consists of a narrow agricultural valley surrounded by seminatural hill moorland. It was initially hypothesized that the higher value at this site was due to site 7 being located at the valley bottom. To investigate such issues of site representativity requires a detailed assessment of local spatial variability. In this case, the 1-km $NH_3$ emission inventory was analyzed with a local dispersion model, combined with ALPHA sampling at 12 locations across the 5-km grid square[24]. This analysis showed that site 7 overestimated the grid average partly due to its location, plus the existence of unexpected local emissions from animal processing by a gamekeeper. In contrast, analysis of the model and measurements showed that the most representative measured concentration was 0.3 µg m$^{-3}$, which was very close to the FRAME estimate. Such local assessment studies are obviously not feasible at more than a few locations. Hence, a robust country scale assessment benefits greatly from analysis of many sites, as in Fig. 5.

Fig. 5 shows that, overall, FRAME provides larger $NH_3$ concentrations than the measurements. There are a number of possible reasons for this, including uncertainties in emissions, vertical diffusion scheme of the model, atmospheric conversion and removal processes, and the possibility that stations are more than the average distance from $NH_3$ sources within each grid square. Most of these points would be expected to be equally possible for all of the sites. However, in the case of emissions, it is possible that the emission factors for different source sector activities are differentially biased, and this will be reflected in the estimates for the different source sector dominated areas. Taking the regression between the measurements and model as the consequence of all the uncertainties, it is therefore of interest if sites dominated by different source sectors fall above or below the line. In the case of sheep-dominated areas, the measurements are larger than the average for all the monitoring sites. Conversely, at all of the nonagricultural-dominated sites, the measurements are less than the average. At most of the cattle-dominated sites, the measurements are also less than the average, while for the mixed and pig plus poultry classes, the sites are distributed evenly. These findings suggest that sheep emissions may have been underestimated and nonagricultural sources and cattle overestimated relative to emissions from pig and poultry. The least certain of these comparisons are the nonagricultural sources, which are much less accurately mapped and include a wide diversity of sources that are not represented as dominant sources here. In addition to these source sector differences, there is also a systematic deviation for the background sites, with three quarters of the sites having larger concentrations than the average relationship. This may partly be due to emissions from wild animals and sheep in these background areas, but is most likely due to the existence of a "compensation point" concentration for $NH_3$ with seminatural vegetation[2,5,6]. This is not represented in the version of FRAME applied here, but is a subject of ongoing model development.

While the correlation for $NH_4^+$ aerosol is much closer than for $NH_3$, it is still substantially above the 1:1 line. This is most likely due to insufficient wet scavenging of $NH_4^+$ aerosol, which is consistent with an underestimation of wet $NH_x$ deposition by FRAME. Again, this is a subject of ongoing model development, where preliminary tests have shown that increasing the scavenging rate gives better agreement with the measurements for both $NH_4^+$ aerosol and $NH_x$ wet deposition. It may be noted that there are no discernible differences for $NH_4^+$ aerosol between the sites dominated by different sectors. This is not surprising, since the $NH_4^+$ concentration responds to $NH_3$ emissions and atmospheric chemistry at a regional level.

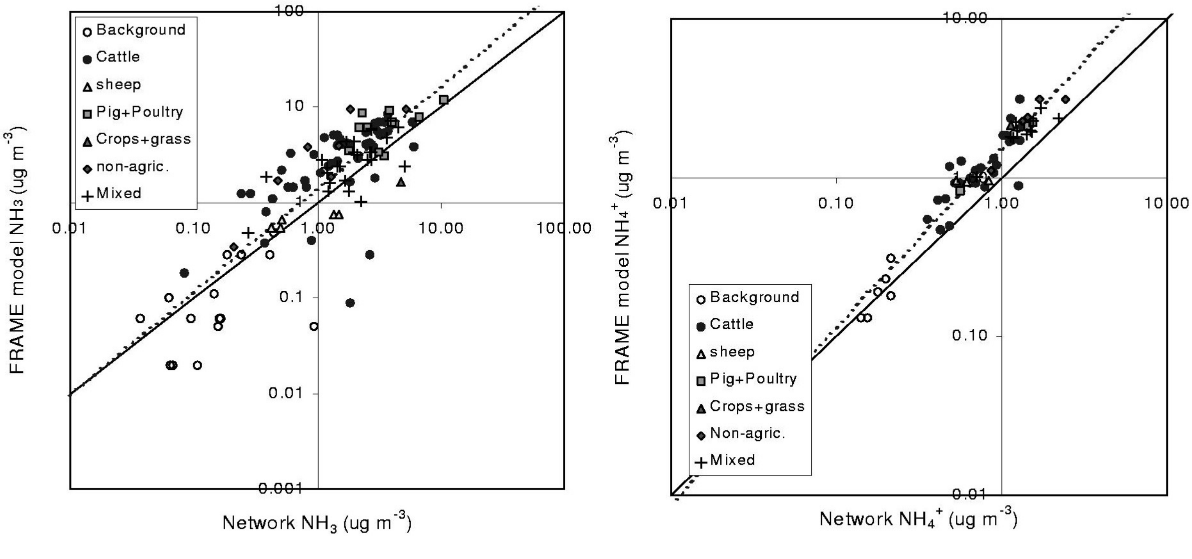

**FIGURE 5.** Comparison of measured $NH_3$ and $NH_4^+$ concentrations in the U.K. with estimates of FRAME classified by estimated dominant $NH_3$ source sector for the 5-km grids in which sampling sites occur. The regressions are: $NH_3$, log(FRAME) = 1.0769 log(NAMN) + 0.1379; $NH_4^+$, log(FRAME) = 1.1179 log(NAMN) + 0.1732.

## Estimation of Dry Deposition

The FRAME model described here incorporates a simplified treatment of dry deposition according to land cover type. To provide more accurate deposition estimates, a more detailed inferential model is applied, which includes treatment of the ammonia compensation point for agricultural vegetation[22]. The regression between the $NH_3$ measurements and FRAME is used to scale the FRAME estimates to the network, and this is applied as input to the inferential model. The outputs of this model are maps of $NH_3$ dry deposition to five major land-cover types: forest, moorland (and low seminatural vegetation), agricultural grassland, arable, and urban. To estimate the budget of U.K. $NH_3$ dry deposition, these maps are combined according to percentage of land-cover occurrence in each 5-km grid-square, which gives a map of grid average $NH_3$ dry deposition. Fig. 6 shows as an example the comparison of the average grid square $NH_3$ dry deposition with that received by moorland/low seminatural vegetation.

Dry deposition $NH_3$ inputs on a grid-square basis are mostly in the range 0 to 10 kg N $ha^{-1}$ $year^{-1}$, which is similar to inputs as $NH_4^+$ in wet deposition. The largest regional dry deposition is estimated for Wales, where relatively large $NH_3$ emissions combine with a large percentage of land cover of moorland and seminatural vegetation. By contrast to the rather small values of average dry deposition, the $NH_3$ dry deposition received by moorland and low seminatural vegetation is much larger, in the range 0 to 40 kg N $ha^{-1}$ $year^{-1}$, and the distribution of this matches more closely to the pattern of estimated $NH_3$ concentrations. It should be noted that Fig. 6 shows the deposition that would be received by low seminatural vegetation if present, which explains why values are provided for the whole of the country.

The distinction between maps of dry deposition to grid squares and to receptors is an important one. While the former are useful to integrate total dry deposition for the country, the latter are what should be applied in summing deposition inputs for comparison with critical loads. The application of the grid average dry deposition field would give a substantial underestimation of critical load exceedance for seminatural vegetation because of the slower rates of $NH_3$ dry deposition to agricultural land. In many locations, the receptor-specific $NH_x$ dry deposition estimates exceed critical loads for nitrogen deposition and acidification, even without counting other atmospheric inputs. It

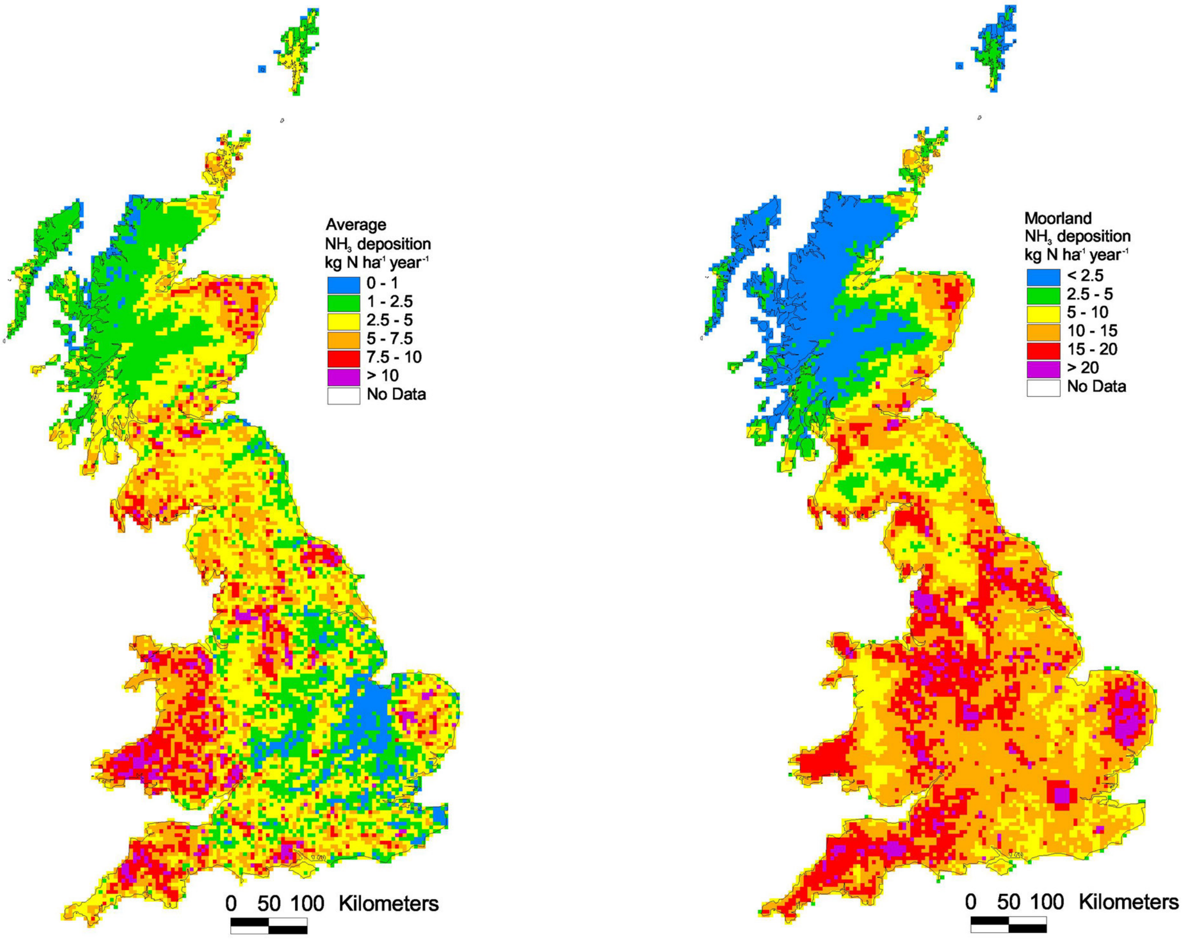

**FIGURE 6.** Estimated dry deposition of ammonia in Great Britain: a) average dry deposition to grid squares, b) dry deposition received by moorland/low seminatural vegetation where present.

is therefore clear that policies to reduce the effects of acidification and eutrophication must include measures to abate $NH_3$ emissions and deposition.

## CONCLUSIONS

A combination of a monitoring network and modeling capability has been established to quantify the spatial patterns of gaseous $NH_3$ and $NH_4^+$ aerosol concentrations and deposition in the U.K. While monitoring of $NH_4^+$ aerosol representatively is possible with only a few tens of sites, the high spatial variability of $NH_3$ would require thousands of monitoring stations to characterize spatial patterns. The variability of $NH_3$ is related to its nature as a primary pollutant emitted in the rural landscape, with the result that meso- and local-scale sampling shows potentially as much variability as at the national scale. Precise site representativity may be investigated using sampling with many sites at a sub-5-km grid level, but is only feasible for a few locations. The U.K. network has therefore been established with many $NH_3$ sampling sites in order to build up a representative picture of $NH_3$ at the U.K. scale. While the sampling methods have been found to give reliable measurements for the full range of concentrations encountered, the use of over 80 sites proves to be adequate to reveal the major spatial differences in $NH_3$ across the U.K.. This large number of sites has the advantage that sites in areas dominated by different $NH_3$ source sectors can also be compared. The analysis here shows that $NH_3$ emissions from sheep may have been underestimated relative to those from cattle, pig, and poultry.

Combining the measurements with estimates from an atmospheric transport model provides the facility to estimate dry deposition inputs at a 5-km grid resolution. This level of detail is necessary since assessment of critical loads exceedance is made at this or even finer (1-km) scales. While maps of interpolated measured $NH_3$ concentrations are useful to envisage the main patterns, they are too uncertain to apply in mapping deposition at a 5-km resolution.

The model of $NH_3$ dry deposition makes the distinction between different land-cover types and simulates the larger inputs characteristic of seminatural compared with agricultural vegetation. This is important, since the receptor-specific estimates for seminatural land may substantially exceed critical loads, even in the absence of other sources of nitrogen deposition. The consequence is that policies to reduce the effects of acidification and eutrophication must include approaches to reduce $NH_3$ emissions and dry deposition. The U.K. National Ammonia Monitoring Network has been established to address spatial patterns with low-frequency (monthly) sampling. It is therefore well-placed to assess the long-term changes in $NH_3$ and $NH_4^+$ following the implementation of emission control policies.

## ACKNOWLEDGMENTS

We gratefully acknowledge funding of the National Ammonia Monitoring Network and of the project “Acid Deposition Processes” by the U.K. Department of Environment, Food and Rural Affairs, together with underpinning funding from the Natural Environment Research Council. We are grateful to Neil Cape and Roger Phillips for their helpful comments on earlier drafts of this article. The Ammonia Network would not be possible without the dedicated input of over 50 local site operators, to whom we express our thanks.

| | | |
|---|---|---|
| Received: | August | 8, 2001 |
| Revised: | October | 10, 2001 |
| Accepted: | October | 15, 2001 |
| Published: | November | 28, 2001 |

## APPENDIX
### Summary of Data from the U.K. National Ammonia Monitoring Network (Netw) and Comparison with the FRAME Model

| Site No | Site Name | National Grid Ref. | Netw. $NH_3$ | FRAME $NH_3$ | Netw. $NH_4^+$ | FRAME $NH_4^+$ | DS |
|---|---|---|---|---|---|---|---|
| 1 | Bush OTC | nt245635 | 2.54 | 2.77 | 0.71 | 1.02 | 5 |
| 2 | Bush Cabin | nt247638 | 1.08 | 2.77 | | | 5 |
| 3 | Inverpolly | nc187088 | 0.07 | 0.02 | 0.16 | 0.13 | 0 |
| 4 | Penallt | so523095 | 1.45 | 2.69 | 1.15 | 1.70 | 1 |
| 5 | Priddy | st525526 | 1.47 | 3.92 | 0.07 | 1.62 | 1 |
| 6 | Holme Lacy | so554357 | 2.14 | 2.91 | 1.29 | 1.70 | 1 |
| 7 | Glen Shee | no117693 | 2.66 | 0.28 | 0.36 | 0.54 | 1 |
| 7b | Gulabin Lodge | no110701 | 1.84 | 0.09 | 0.43 | 0.47 | 1 |
| 8 | Stackpole | sr982947 | 2.26 | 1.04 | | | 6 |
| 8b | Orielton | sr954992 | 1.30 | 2.56 | 0.74 | 1.15 | 1 |
| 9 | Brown Moss | sj559396 | 5.94 | 6.91 | | | 1 |
| 9b | Brown Moss 2 | sj563390 | 3.50 | 6.91 | 1.15 | 2.36 | 1 |
| 10 | Bure Marshes | tg334161 | 1.21 | 2.33 | 1.24 | 1.98 | 6 |
| 11 | Mere Sands Wd. | sd447157 | 1.98 | 3.33 | 1.40 | 1.89 | 6 |
| 12 | Halladale | nc902488 | 0.94 | 0.05 | 0.21 | 0.18 | 0 |
| 13 | Aston Rowant. | su727979 | 1.69 | 1.7 | 1.54 | 2.26 | 6 |
| 14 | Ellon Ythan | nj945304 | 1.53 | 4.64 | 0.46 | 0.74 | 1 |
| 15 | Llynclys Comn. | sj273237 | 1.34 | 5.01 | 1.07 | 1.85 | 1 |
| 16 | Northallerton | se360930 | 3.66 | 4.75 | 1.51 | 1.94 | 6 |
| 17 | Easingwold | se540675 | 4.52 | 6.06 | 1.22 | 2.22 | 6 |
| 18 | Auchencorth | nt221562 | 0.81 | 1.45 | 0.71 | 1.12 | 1 |

**APPENDIX (CONTINUED)**
**Summary of Data from the U.K. National Ammonia Monitoring Network (Netw) and Comparison with the FRAME Model**

| Site No | Site Name | National Grid Ref. | Netw. $NH_3$ | FRAME $NH_3$ | Netw. $NH_4^+$ | FRAME $NH_4^+$ | DS |
|---|---|---|---|---|---|---|---|
| 19 | Shetland | hu500400 | 0.15 | 0.11 | 0.20 | 0.23 | 0 |
| 20 | Drayton | sp165549 | 3.46 | 3.09 | 1.45 | 2.12 | 2 |
| 21 | Glensaugh | no664799 | 0.29 | 1.24 | 0.52 | 0.92 | 1 |
| 22 | Moor House | ny751334 | 0.24 | 0.28 | 0.64 | 0.92 | 0 |
| 23 | North Wyke | sx659983 | 1.71 | 4.15 | 0.93 | 1.20 | 1 |
| 24 | Rothamsted | tl123129 | 1.29 | 1.84 | 1.34 | 2.26 | 5 |
| 25 | Sourhope | nt867218 | 0.51 | 0.55 | 0.53 | 0.95 | 3 |
| 26 | Wytham Woods | sp452083 | 1.21 | 2.03 | 1.21 | 2.24 | 6 |
| 27 | Alice Holt | su809379 | 1.24 | 1.58 | 1.14 | 1.81 | 6 |
| 28 | Porton Down | su253365 | 1.72 | 4.34 | 1.18 | 1.78 | 6 |
| 29 | Llydaw | sh695572 | 1.36 | 0.74 | 0.62 | 0.94 | 3 |
| 30 | Strathvaich Dm. | nh348750 | 0.11 | 0.02 | 0.18 | 0.19 | 0 |
| 31 | Eskdalemuir | nt235030 | 0.37 | 0.37 | 0.56 | 0.92 | 1 |
| 32 | High Muffles | se776939 | 1.84 | 1.64 | 1.01 | 1.83 | 1 |
| 33 | Stoke Ferry | tl700988 | 2.19 | 6.05 | 1.56 | 2.21 | 2 |
| 34 | Yarner Wood | sx789788 | 0.52 | 2.15 | 0.90 | 1.08 | 1 |
| 35 | Lullington Hth. | tq538016 | 0.64 | 1.44 | 1.29 | 3.10 | 1 |
| 36b | London, Vict. | tq291790 | 1.85 | 9.5 | 1.69 | 3.10 | 5 |
| 36c | London, Crom. | tq266791 | 5.25 | 9.5 | 2.42 | 3.10 | 5 |
| 37 | 5 Acres | sw794486 | 0.93 | 3.16 | 1.27 | 0.89 | 1 |
| 38 | Sheffield | sk332870 | 0.84 | 3.8 | 1.44 | 2.39 | 5 |
| 39 | Silsoe | tl088356 | 1.51 | 2.42 | 1.49 | 2.29 | 6 |
| 40 | Sutton Bonntn. | sk505268 | 5.15 | 2.41 | 1.72 | 2.75 | 6 |
| 41 | Lagganlia | nh856037 | 0.16 | 0.06 | 0.21 | 0.31 | 0 |
| 42 | Castle Cary | st609319 | 3.69 | 5.53 | 1.12 | 1.67 | 1 |
| 43 | Tadcaster | se452455 | 2.10 | 3.15 | 1.22 | 2.21 | 6 |
| 44 | Hillsborough | ij243577 | 3.22 | 5.07 | 0.64 | 1.00 | 1 |
| 45 | Lough Navar | ih065545 | 0.43 | 1.08 | 0.41 | 0.72 | 1 |
| 46 | Sibton | tm363722 | 2.67 | 3.4 | 1.49 | 2.00 | 6 |
| 47 | Rum | nm408992 | 0.16 | 0.05 | 0.14 | 0.13 | 0 |
| 48 | Wem Moss | sj473343 | 2.91 | 6.77 | | | 1 |
| 49 | Frodsam | sj525795 | 3.17 | 3.4 | | | 2 |
| 50 | Swettenham Mds. | sj804674 | 3.52 | 5.4 | | | 1 |
| 51 | Wybunbury Moss | sj698502 | 3.06 | 5.59 | | | 1 |
| 52a | Fenn's Moss 1 | sj490365 | 2.48 | 4.03 | | | 1 |
| 52b | Fenn's Moss 2 | sj478368 | 1.85 | 4.03 | | | 1 |
| 52c | Fenn's Moss 3 | sj498378 | 1.60 | 4.03 | | | 1 |
| 53 | Little Budworth | sj584658 | 2.71 | 2.82 | | | 6 |
| 54 | Bickerton Hill | sj498527 | 2.67 | 4.26 | | | 1 |
| 55 | Ruabon | sj225489 | 0.58 | 1.44 | | | 5 |
| 56 | Wardlow Hay Cop | sk177737 | 1.22 | 2.42 | | | 1 |
| 57 | Stanford | tl858948 | 1.81 | 3.47 | | | 2 |
| 58 | Redgrave+Lophm. | tm050797 | 2.76 | 5.71 | | | 6 |
| 59 | Dunwich Heath | tm470680 | 1.23 | 1.32 | | | 6 |
| 60 | Edinburgh | nt253734 | 1.49 | 3.97 | 0.87 | 1.11 | 5 |

**APPENDIX (Continued)**
**Summary of Data from the U.K. National Ammonia Monitoring Network (Netw) and Comparison with the FRAME Model**

| Site No | Site Name | National Grid Ref. | Netw. $NH_3$ | FRAME $NH_3$ | Netw. $NH_4^+$ | FRAME $NH_4^+$ | DS |
|---|---|---|---|---|---|---|---|
| 61 | Much Hoole | sd473231 | 3.87 | 9.31 | | | 2 |
| 62 | Midge Hall | sd508231 | 2.72 | 5.98 | | | 6 |
| 63 | Cardigan | sn185453 | 0.62 | 3.32 | 0.48 | 1.17 | 1 |
| 64 | Pwllpeiran | sn798771 | 0.51 | 0.66 | | | 3 |
| 65 | Allt a Mharcaidh | nh895024 | 0.09 | 0.06 | | | 0 |
| 66 | Dennington | tm276669 | 4.05 | 7.05 | | | 2 |
| 67 | Fressingfield | tm261759 | 6.63 | 7.87 | | | 2 |
| 68 | Bedlingfield | tm173684 | 10.66 | 11.99 | | | 2 |
| 70 | Cwmystwyth | sn771742 | 1.48 | 0.74 | 0.84 | 0.96 | 3 |
| 71 | Myerscough | sd498399 | 3.73 | 8.27 | | | 1 |
| 72 | Cardoun Burn | nx546658 | 0.19 | 0.28 | | | 0 |
| 73 | Jenny Hurn | sk816986 | 1.80 | 1.31 | 2.23 | 2.34 | 6 |
| 74 | Carlisle | ny468554 | 3.34 | 5.11 | 0.90 | 1.32 | 1 |
| 75 | Westhay Moor | st455440 | 1.15 | 4.81 | | | 1 |
| 76 | Pointon | tf128313 | 4.77 | 1.64 | 1.13 | 2.14 | 4 |
| 77 | Carradale | nr798378 | 0.89 | 0.40108 | 0.48 | 0.50 | 1 |
| 78 | Auchincruive | ns379234 | 6.03 | 3.79 | 0.69 | 0.93 | 1 |
| 78b | Auchincruive 2 | ns384229 | 2.87 | 3.79 | | | 1 |
| 79 | Coleraine | iC884211 | 2.49 | 5.42 | 0.78 | 0.87 | 1 |
| 80 | Lyulphs Tower | ny403202 | 0.80 | 1.69 | 0.57 | 1.28 | 1 |
| 81 | Pitmedden | nj883278 | 2.32 | 8.59 | 0.56 | 0.82 | 2 |
| 82 | Brompton | se389988 | 3.92 | 7.06 | | | 6 |
| 83 | Barcombe Mills | tq438149 | 1.05 | 2.02 | 1.34 | 2.11 | 1 |
| 84 | Thursley Common | su910404 | 0.39 | 1.86 | | | 6 |
| 85 | Savarnake | su055888 | 2.90 | 1.81 | | | 1 |
| 86 | Lakes | sd337941 | 0.39 | 0.8 | | | 1 |
| 87 | Thetford | tl944841 | 1.98 | 4.38 | | | 6 |
| 88 | Sherwood | sk163905 | 0.45 | 0.49 | | | 0 |
| 89 | Rannoch | nn603533 | 0.04 | 0.06 | | | 0 |
| 90 | Coalburn | ny693782 | 0.21 | 0.34 | | | 5 |
| 91 | Tummel | nn744611 | 0.06 | 0.1 | | | 5 |
| 92 | Loch Awe | nm966115 | 0.08 | 0.18 | | | 1 |
| 93 | Llynn Brianne | sn816484 | 0.27 | 0.48 | | | 6 |
| 94 | Alice Holt (2) | su805427 | 0.48 | 1.7 | | | 5 |

*Note:* At some sites, more than one independent measurement system is applied (not shown here). Values in $\mu g\ m^{-3}$. DS = dominant source sector: 0, background; 1, cattle; 2, pig + poultry; 3, sheep; 4, crops + grass; 5, nonagricultural; 6, mixed.
